# Yttrium-90 trans-arterial radioembolization in advanced-stage HCC: The impact of portal vein thrombosis on survival

**DOI:** 10.1371/journal.pone.0216935

**Published:** 2019-05-29

**Authors:** Francesco Somma, Vincenzo Stoia, Nicola Serra, Roberto D’Angelo, Gianluca Gatta, Francesco Fiore

**Affiliations:** Dipartimento di Radiologia Interventistica, Istituto Nazionale Tumori IRCCS “Fondazione G. Pascale”, Napoli, Italy; Northwestern University Feinberg School of Medicine, UNITED STATES

## Abstract

**Purpose:**

Portal vein thrombosis (PVT) is generally recognized as a prognostic factor in HCC. Our purpose is to assess and compare the survival of patients with PVT and without PVT, after Y-90 Trans-Arterial Radio-Embolization (TARE) of unresectable HCC, unresponsive to other loco-regional treatments.

**Materials and methods:**

Between November 2005 and November 2012, Y-90 resin-based TARE was performed in an IRB-approved prospective protocol, on 89 patients with unresectable HCC. 33/89 patients had PVT, the remaining 56 were resistant-to-cTACE or underwent TARE as a downstaging therapy. All patients were studied with Multi-Detector Computed Tomography (MDCT), angiography, ^99m^Tc-MAA-scintigraphy and liver biopsy. Gastro-duodenal artery was embolized in most cases. Proton-Pump Inhibitors were administered to prevent gastritis and ulcers. χ^2^ test with Yates correction and log rank test were used to compare the two proportions and Kaplan-Meier survival curves, respectively.

**Results:**

The average activity administered was 1.7 ± 0.4 GBq. After the treatment, CTCAE grade 2 adverse events occurred in 46% (41/89) patients: in particular, fever and abdominal pain were found in 25 and 16 patients, respectively. No major side-effect was observed. According to mRECIST criteria, partial response or complete response was found in 70% of patient three months after the treatment, and in 90.5% nine months after the treatment. No significant difference was found in survival of patients with PVT compared to those without PVT (p-value = 0.672). A complete regression of PVT was observed in almost half patients (13/27, 48.1%).

**Conclusions:**

Portal vein invasion does not affect survival in advanced stage HCC-patients undergoing TARE using Y-90 resin-based microspheres. Y90 procedure is associated with regression of portal vein tumor thrombus.

## Introduction

Portal Venous Thrombosis (PVT) is a common complication of Hepatocellular Carcinoma (HCC), assessed as adverse prognostic factor and parameter of tumor aggressiveness together with tumor size, multifocality and Alpha-fetoprotein (AFP) levels [1,2]. Approximately 10%-40% patients with HCC have PVT at the time of diagnosis [3], and 35%-44% will be found to have PVT at the time of death or liver transplant [4]. Patients with PVT are more likely to have metastatic disease at diagnosis and a shortened overall survival compared to patients without PVT. Thrombus involving the main portal vein is a worse prognostic factor than thrombus involving a branch portal vein [5]. Curative treatments (resection, transplantation and percutaneous ablation) are not generally indicated in patients with PVT [6,7], since the majority show an intermediate-to-advanced stage disease at presentation [8]. According to Barcelona Clinic Liver Cancer (BCLC), sorafenib and Trans-arterial Chemo-embolization (TACE) are recommended as the standard of care for patients with intermediate-advanced stage HCC: however, sorafenib has been shown only to modestly prolong survival, while TACE is generally regarded as contraindicated in patients with PVT, due to the higher risk of complications, including acute liver failure or intrahepatic tumor progression [9,10]. In this scenario, Trans-arterial Radio-embolization (TARE) has gained increasing awareness and usage within the last decade, showing to be efficient in down-staging advanced HCC before resection or transplantation, even in case of PVT [11].

The aim of this study is to assess and compare survival in HCC patients with PVT and without PVT, after Y90 resin-based TARE for the treatment of unresectable HCC, unresponsive to other loco-regional treatments.

## Material and methods

### Ethics statements

This study has been approved by the Institutional Scientific Committee and Review Board of the National Cancer Institute “Comitato Etico IRCCS Fondazione Pascale” (Napoli). Appropriate written informed consent was collected before all procedures.

### Patient population

Between November 2005 and November 2012, 102 unresectable HCC were proposed for Yttrium-90 (Y-90) TARE in our Interventional Radiology Department by a multi-disciplinary team. Inclusion criteria were: advanced HCC, hepatic disease volume ≤50% of total liver volume, HCC confirmed by liver biopsy. Exclusion criteria were: hepatic insufficiency (bilirubin value ≥2.6 mg/dl; Child-Pugh score ≥9); Eastern Cooperative Oncology Group (ECOG) ≥2); life-expectancy inferior to 3 months; massive extra-hepatic spreading disease.

Base in the above reported criteria, 14 patients were excluded. The remaining 89 patients (55.56% male; 44.44% female; range of age 36-86years) underwent TARE. The following patient characteristics were found: lesion-size 1.1-to-12.3cm; Child-Pugh score 5-to-8; bilirubine values up to 2.5mg/dl. 33 patients had PVT, while the remaining 56 were elderly patients, and/or resistant to other trans-arterial modalities such as conventional TACE (cTACE) and Trans-arterial Ethanol-Lipiodol Embolization (TAELE) [12], or underwent TARE as a pre-resection down-staging treatment.

Abdominal images were reviewed by a 20-year liver experienced radiologist, blinded to the therapy performed. Prior radical and non-radical treatments were listed in Table **1**.

**Table 1:** Baseline (T_0_) demographics for enrolled patients.

Parameters	Portal Invasion		
	None [56/89]	Peripheral branch [22/89]	Main trunk [11/89]
***Age*** (y)			
<65 [37/89]	21/56 (37.50)	11/22 (50,00)	5/11 (45,45)
≥65 [52/89]	35/56 (62.50)	11/22 (50,00)	6/11 (54,55)
***Sex***			
Male [53/89]	31/56 (55.36)	15/22 (68,18)	7/11 (63,63)
Female [36/89]	25/56 (44.64)	7/22 (31,82)	4/11 (36,37)
***Cause***			
Hepatitis C virus [69/89]	43/56 (76.78)	16/22 (72,72)	10/11 (90,90)
Hepatitis B virus [7/89]	5/56 (8.93)	1/22 (4,55)	1/11 (9,10)
Alcohol [6/89]	3/56 (5.36)	3/22 (13,64)	0/11 (0,00)
Cryptogenetic [5/89]	3/56 (5.36)	2/22 (9,09)	0/11 (0,00)
NASH [2/89]	2/56 (3.57)	0/22 (0,00)	0/11 (0,00)
***Prior Radical Treatment***			
None [54/89]	35/56 (62,50)	10/22 (45,45)	9/11 (81,82)
RFA [27/89]	15/56 (26,78)	11/22 (50,00)	1/11 (9,09)
Wide resection [7/89]	6/56 (10,72)	1/22 (4,55)	1/11 (9,09)
***Prior Vascular/Percutaneous Treatment***			
None [47/89]	37/56 (66,07)	7/22 (31,82)	3/11 (27,27)
TACE [16/89]	9/56 (16,07)	4/22 (18,18)	3/11 (27,27)
TAELE [18/89]	6/56 (10,71)	8/22 (36,36)	4/11 (36,36)
RFA [7/89]	4/56 (7,14)	2/22 (9,09)	1/11 (9,10)
TARE [1/89]	0/56 (0,00)	1/22 (4,55)	0/11 (0,00)

### Pre-treatment assessment

Patient evaluation and staging was assessed on the base of demographics, aetiology, risk factors, biopsy results, comorbidities, presence or absence of cirrhosis and PVT. Imaging was performed by Multi Detector Computed Tomography (MDCT) before and after intravenous contrast medium administration using a dual-phase protocol with arterial and portal phases. Underlying liver disease was determined according to Child–Pugh, Tumor-Node-Metastasis (TNM) and BCLC classifications. ECOG was also used to asses the patients’ Performance Status (PS) and how the disease affected the daily living abilities of the patient. Among all patients classified as ECOG PS >0 at baseline, only those showing cancer-related symptoms were actually classified as BCLC C.

Within 4 weeks before treatment, patients underwent an evaluation including: chest and abdominal multi-phase MDCT to measure the volume of the total tumor mass. Complete blood count, liver function tests, creatinine, albumin, prothrombin activity, and AFP were monitored.

### TARE procedure

SIR-Spheres (Sirtex, Lane Cove, Australia) was used in all patients. This is an implantable medical device consisting of resin-based, biocompatible microspheres approximately 35μ in diameter loaded with Y90, that is a beta-emitter with a half-life of approximately 64hrs and an average penetration in tissues around 2.4mm [13]. Dosimetry for this therapy has already been discussed in previous published studies [13].

To reduce the risk of non-target embolization, all patients underwent pre-treatment mesenteric angiography and Technetium-99 m-labelled macro-aggregated albumin (^99m^Tc-MAA) scintigraphy few hours before treatment [14–17]. The tumor was approached under fluoroscopic guidance; the activity vial was injected into the vessels feeding the tumor. Tumor distribution guided for the selectivity of the treatment, i.e., to one or more lobes/segments was required. TARE was performed as an outpatient procedure and patients were discharged 4-6hrs after the treatment [17]. Due to the possibility of gastro-intestinal ulcers, prophylactic proton pump inhibitors (PPI) were used for the following 14 days.

All procedures were realized by advancing a micro-catheter (Progreat and Progreat Omega, Terumo Corporation, Tokyo, Japan) with a 2.4-F tip or a 2.7-F tip through a 4-F and 5-F catheter (Imager II C1-Selective and RC2-Selective, Boston Scientific Corporation, Natick, MA, USA), into the branches of the hepatic arteries. A super-selective catheterization of the tumor-feeding arteries was attempted in all cases.

A previous comprehensive angiogram was performed aiming to i) detect variants of arterial liver irrigation, ii) identify vessels giving arterial blood supply to every liver tumor nodule, iii) assess portal vein blood flow, and iv) detect collaterals to the gastrointestinal tract or other extrahepatic organs. After this study, the gastro-duodenal artery and/or other vessels feeding gastrointestinal tract and pancreas were occluded using coils, if necessary. With the tip of the catheter placed in the position intended for microspheres injection, ^99m^Tc-MAA were injected to measure the degree of intrahepatic/intratumoral shunt to the lung, to detect any possible misplacement of microspheres in the gastrointestinal tract, and to evaluate the relative amount of activity going to the liver tumors and the non-tumoral liver [18]. In case of bilobar spreading tumors, TARE was repeated more times and/or associated to other loco-regional treatments.

The radiation activity to administer was calculated by the *Body Surface Area (BSA) model*, using the formula: Activity (GBq) = (BSA—0.2) + (tumor volume/total liver volume) [19]. Tumor liver involvement was measured on MDCT. The calculated activity was reduced for patients with lung shunt of 10% to 20%, and treatment was contraindicated if the lung shunt was higher than 20%. The prescribed dose of Y90 was entirely injected in all patients with no sign of vascular stasis in the lesion. After treatment, patients remained at hospital overnight. 4-week supportive therapy generally consisted of prophylaxis of gastritis (proton pump inhibitor), low-dose of methyl-prednisolone and anti-emetics and analgesics on-demand.

### Response evaluation and toxicity

Radiological response was evaluated according to modified Response Evaluation Criteria in Solid Tumors (mRECIST) and European Association for the Study of the Liver (EASL) guidelines [20,21], using a contrast-enhanced MDCT in dual phase acquisition (arterial phase and venous phase) [22]. The lesion degree of vascularization was evaluated by comparing contrast-enhanced MDCT imaging at baseline and one month after the procedure [23]. Furthermore, laboratory data including serum total bilirubin (normal range 0.2–1.1 mg/mL), aspartate aminotransferase (AST, normal range: 10–40 U/L), alanine aminotransferase (ALT, normal range 10–40 U/L) were examined 1 day before the procedure, 2-, 4-, 6- and 30 days after the procedure in order to monitor the liver toxicity of each procedure. Safety was assessed considering the frequency of adverse events up to 4 weeks following the treatment according to National Cancer Institute’s Common Terminology Criteria for adverse events (CTCAE, version 4.0, 2009). The following were considered as adverse events: Grade 1—abdominal pain, nausea, vomiting, fatigue and fever; Grade 2—thrombocytopenia, leukocytosis, transient increase in liver enzymes, and LDH; Grade 3—acute renal failure, hypoxia, remarkable increase in serum total bilirubin and liver enzymes, severe arterial hypertension; Grade 4—life-threatening consequences of the procedure with urgent operative intervention indicated; Grade 5—death.

### Statistical analysis

The statistical analyses were performed using Matlab statistical toolbox version 2008 (MathWorks, Natick, MA, USA) for Windows at 32 bit, on a random sample of 89 patients, comparing different sub-groups with different characteristics. The following time-points were fixed to ease the statistical estimation of survivals rates: T_0_ –baseline time, immediately before the procedure; T1–3-month follow up; T_2_−6-month follow up; T_3_−12-month follow up; T_4_−24-month follow up; T_5_−36-month follow up. χ^2^ test with Yates correction [24] was used to compare two proportions, and log rank test [25] to compare Kaplan-Meier survival curves [26].

All statistical tests with p-value < 0.05 were considered as significant.

## Results

The population baseline characteristics are listed in Table1. The average activity administered was 1.7 ± 0.4 GBq. The procedure itself was well tolerated, with mild-to-moderate nausea and/or vomiting, abdominal pain and/or fever occurring in less than one-third of patients (27/89, 30.3%). As expected, in patients with underlying chronic liver disease, many patients had grade 1 or 2 abnormal values in liver associated parameters such as INR, bilirubin, platelets and alanine aminotransferase prior to TARE, with no significant change in grade during a 3-months follow-up. Fever and abdominal pain were found in 25 and 16 patients, respectively. According to CTCAE, no adverse event listed in the toxicity grade 4–5 was observed.

According to mRECIST criteria, partial response or complete response was found in 70% (62/89) of patients three months after the treatment, and in 90.5% (80/89) nine months after the treatment.

**Fig 1:**
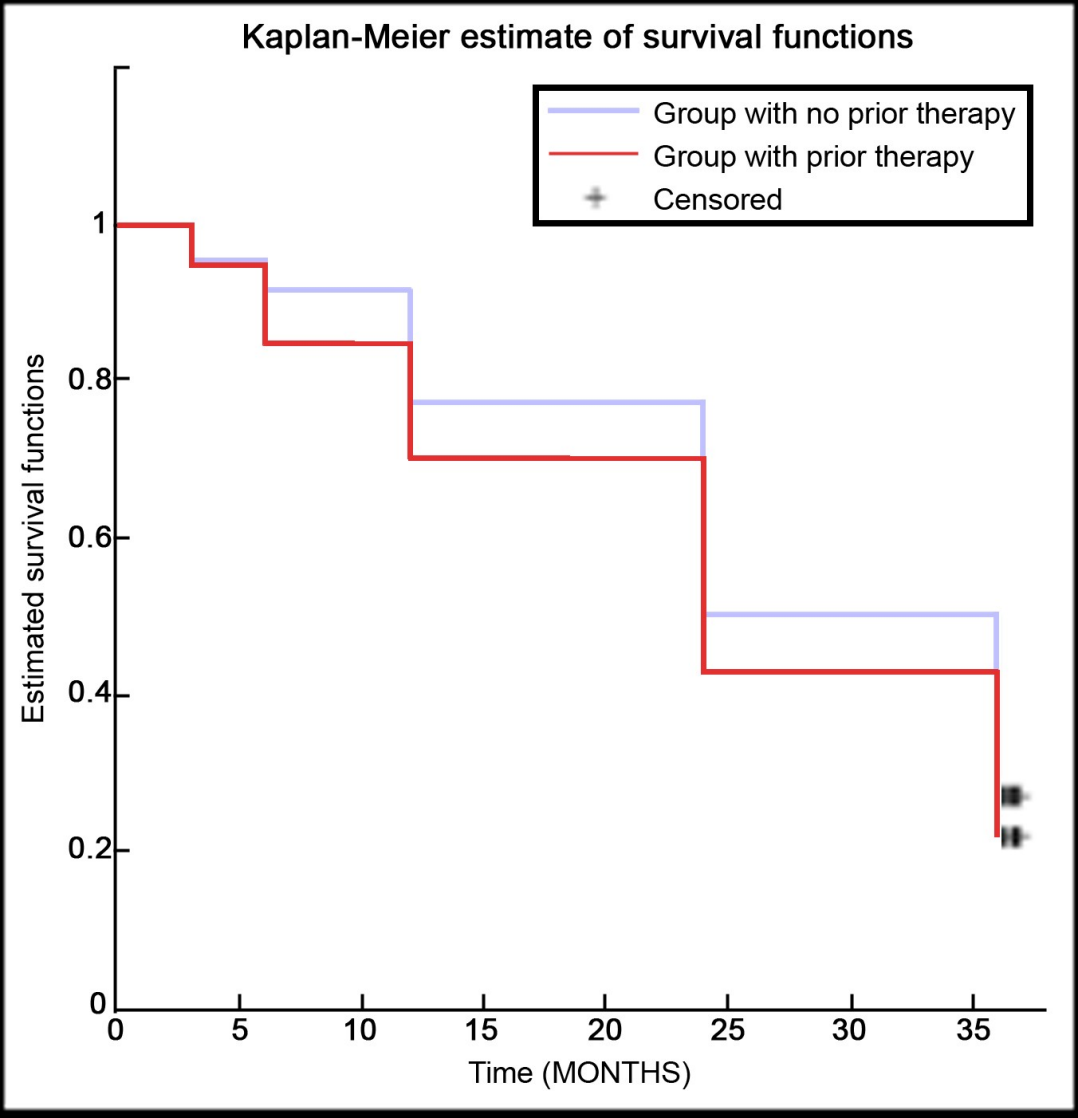
Kaplan-Meier survival curves comparison of ‘naïve’ patients vs. patients with previous treatment.

First of all, patients were divided in those who had undergone a previous loco-regional treatment and those who underwent TARE as first loco-regional procedure. No significant difference was found in terms of survival during 36-month follow-up between Kaplan-Meier survival curves of the two groups (Fig 1), using the log-rank test (p-value = 0.550).

**Fig 2:**
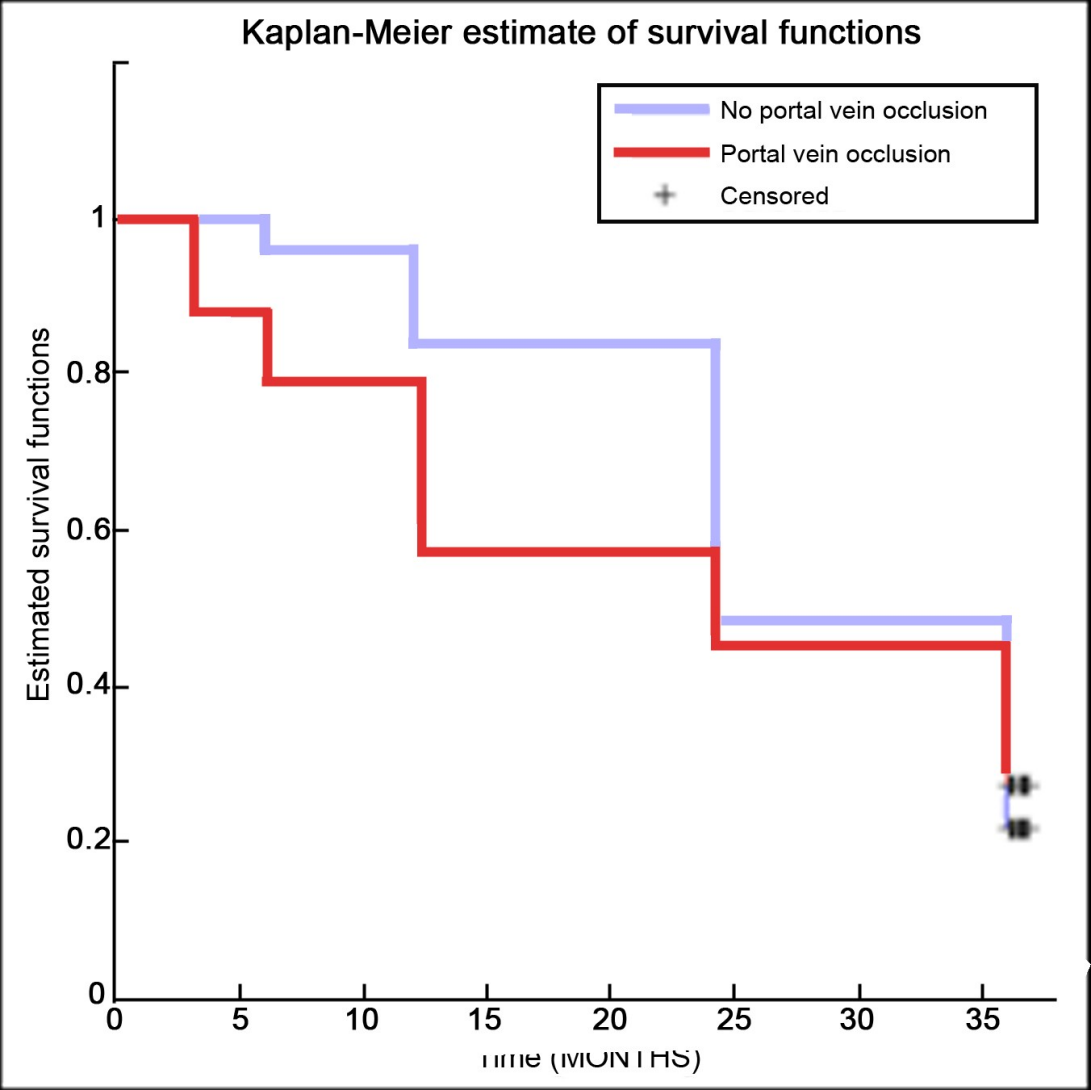
Kaplan-Meier survival curves comparison of patients without portal thrombosis and those with portal vein thrombosis, whether or not the obstruction was in the main trunk or in a peripheral branch.

Secondly, patients were divided on the base of macroscopic portal vein invasion. In particular, patients showing no PVT were compared to those showing portal vein obstruction, whether or not the obstruction was in the main trunk or in a peripheral branch. Even in this case, survival during the 36-month follow-up was estimated for both groups and Kaplan-Meier survival curves were compared (Fig 2), without any significant difference using the log-rank test (p-value = 0.672).

**Fig 3:**
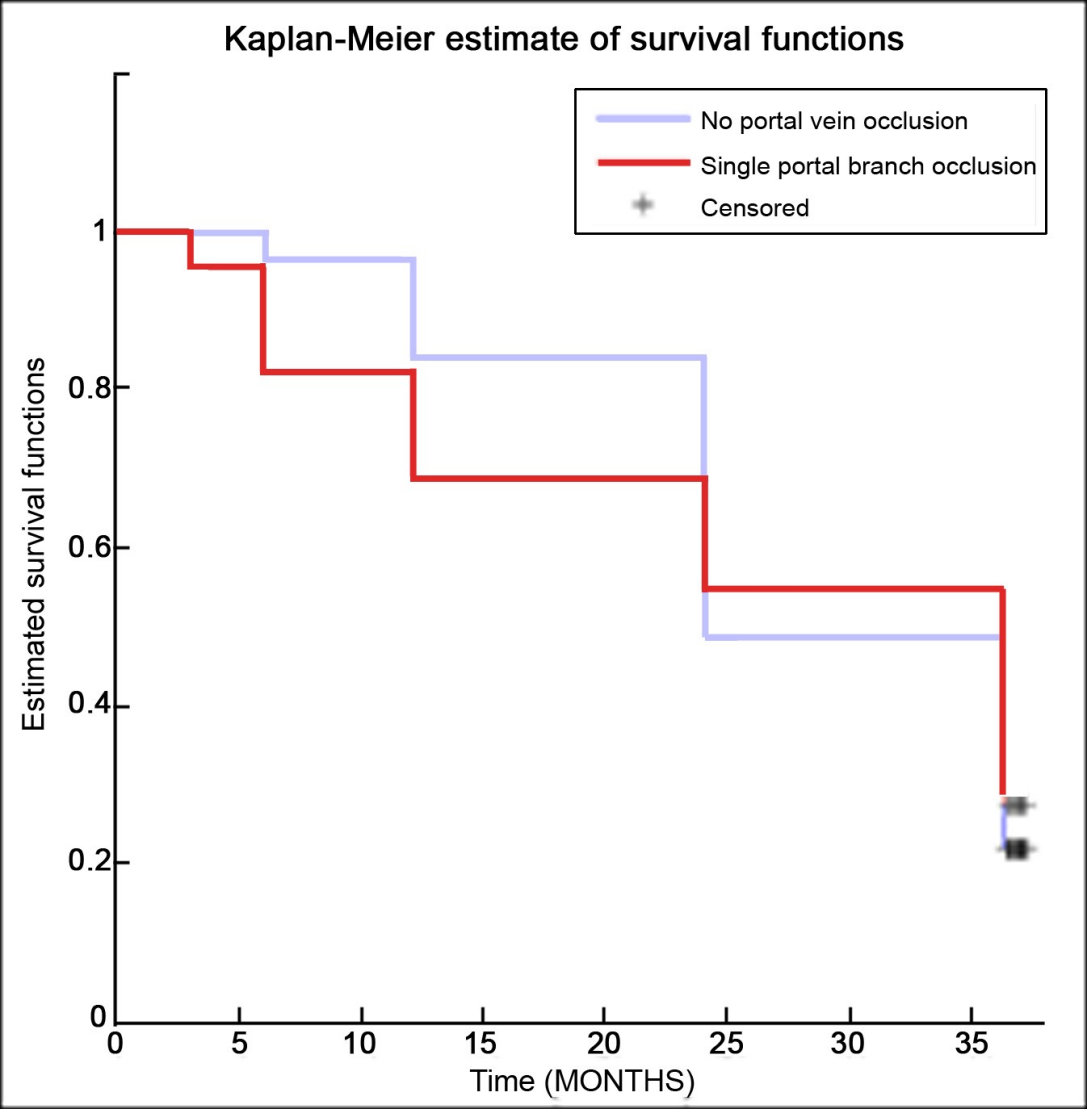
Kaplan-Meier survival curves comparison of patients without portal thrombosis and those with peripheral portal thrombosis.

Following, survival was estimated for patients with only portal branch thrombosis versus those with main portal vein occlusion. Comparing the Kaplan-Meier survival curves (Fig 3), no significant difference was found using the log-rank test (p-value = 0.905).

**Fig 4:**
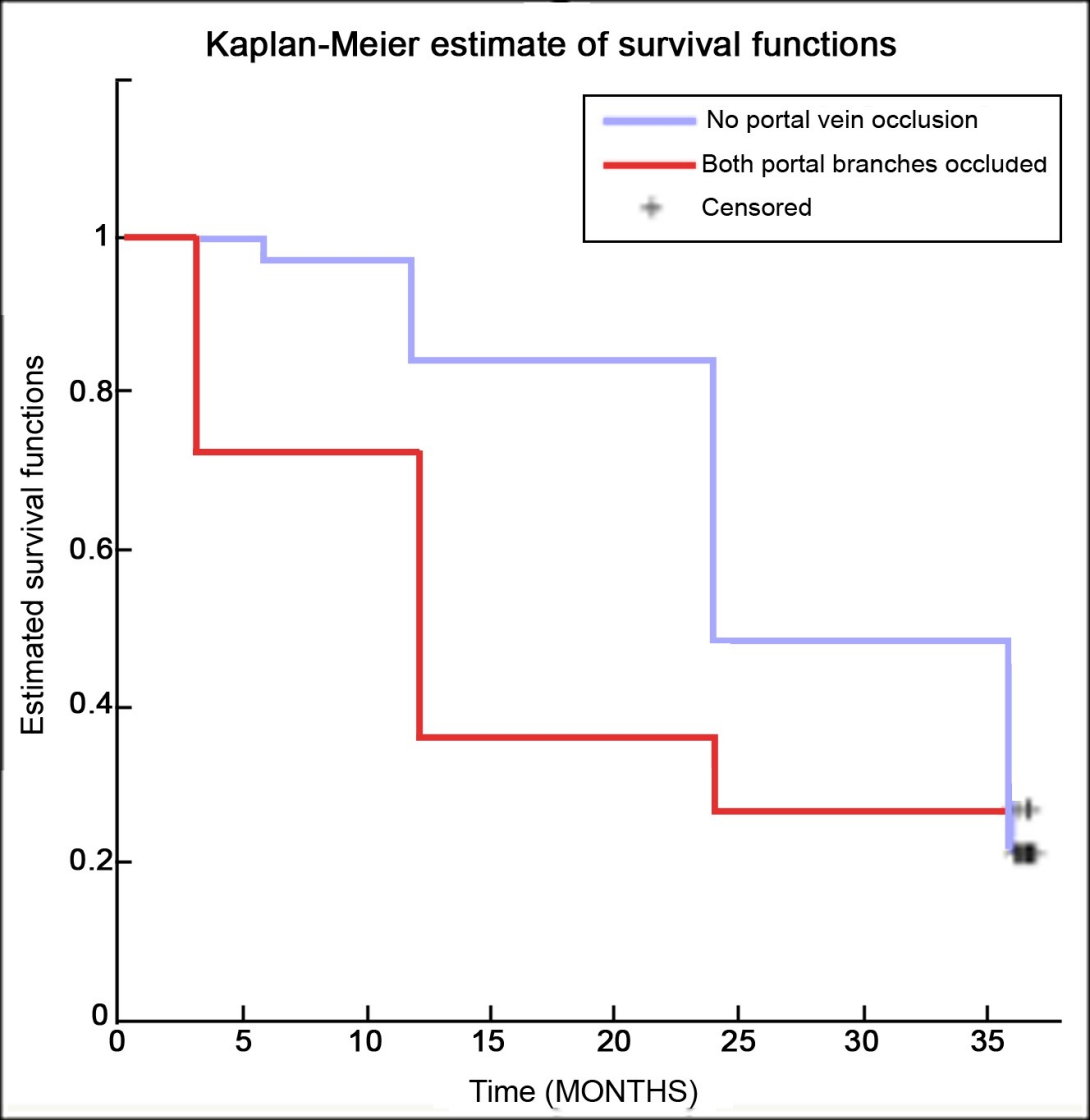
Kaplan-Meier survival curves comparison of patients without portal thrombosis and those with main trunk portal thrombosis.

Similarly, no significant statistical difference was observed in comparing Kaplan-Meier survival curves of patients with no portal vein obstruction and those with main portal trunk thrombosis with p-value = 0.225 according to the log-rank test (Fig 4).

**Fig 5:**
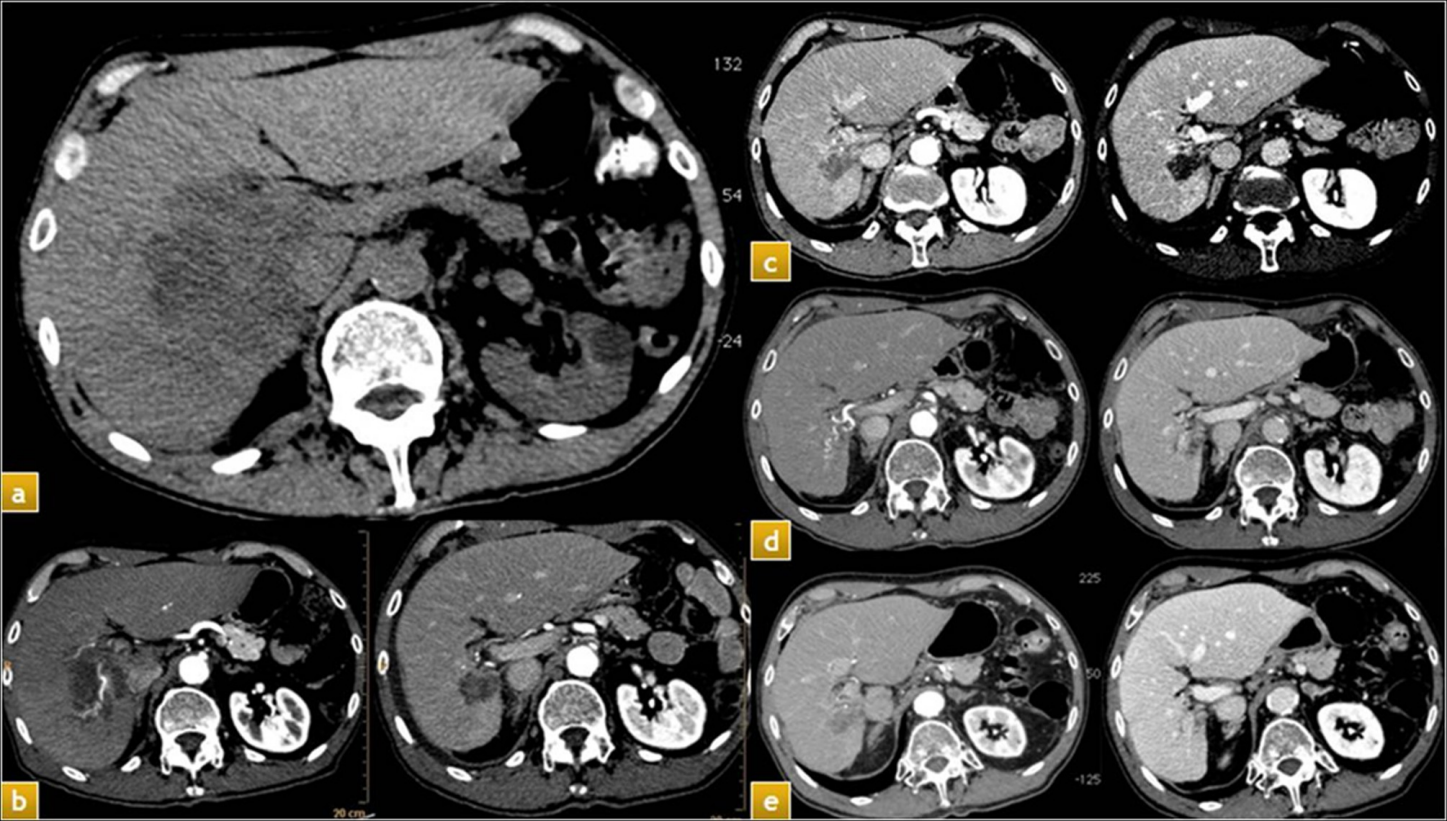
Patient with large HCC in the right lobe with main trunk portal thrombosis: a) baseline CT; b) CT assessment imaging one month after TARE; c) CT imaging six months after TARE; d) CT imaging 12 months after TARE; e) CT imaging 24 months after TARE.

Eventually, the persistence of tumor portal thrombosis was investigated in patients showing portal obstruction at T_0_. All portal thrombus were reputed as tumor on the basis of imaging. An overall regression of PVT was found in almost half patients alive at T_2_ (13/27, 48.1%, e.g. Fig 5), with the maximum effect in retracting portal thrombus in the time-range T_0_-T_2_ (12/13, 92.3%), while only 1 patient showed thrombosis regression in the time-range T_0_-T_1_ (1/13, 7.7%). No further significant evolution of portal thrombosis was observed after T_2_.

## Discussion

HCC is a condition in which many patients present beyond potentially curative options [27]. PVT represents a complication in up to 40% of HCC at presentation, and is associated with a poor prognosis. In case of supportive care, overall survival ranges from two to four months, compared to 10–24 months in HCC patients without PVT [3].

Transplantation is generally regarded as contraindicated in HCC with PVT due to high rates of tumor recurrence [28]. Surgical resection is often technically infeasible and associated with poorer outcomes [29]. Radio-Frequency Ablation (RFA) is potentially curative only for small tumors without portal invasion [30]. Sorafenib is currently the only systemic therapy specifically recommended for HCC with PVT in American Association for the Study of Liver Disease (AASLD) and EASL guidelines [31,32]. It was the first systemic agent shown to extend overall survival in patients with unresectable HCC, including those with PVT [33–35]. The Sorafenib HCC Assessment Randomized Protocol (SHARP) trial [33] compared sorafenib to placebo in Child-Pugh A patients with advanced-HCC showing median survival of 10.7mo compared to 7.9mo in the control group. Although a selected group of patients responds remarkably to sorafenib, even to the point of downstaging [36–38], the majority of patients with PVT have relatively short overall survival expectancy despite treatment [37,38], which has inspired continued efforts at developing loco-regional therapeutic options.

Palliation with cTACE (doxorubicin-oil emulsion followed by gelatine sponge) showed survival improvement when compared with best supportive care [39]. Therefore, cTACE has become the trans-arterial standard of care for unresectable HCC patients [40,41]. Recently, a drug-eluting bead (DC-Bead) has been developed to enhance tumor drug delivery and reduce systemic availability. A randomized trial by Lammer et al (PRECISION V) [42] failed to prove the superiority of DC-Bead TACE over cTACE in patients without PVT. TACE has an established role of loco-regional therapy for inoperable tumors, even as a means of maintaining local control of tumor while awaiting definitive surgical management (the so-called “bridge-to-transplant”) [32]. Historically, PVT has been considered a contraindication to TACE due to the risk of precipitating liver necrosis and worsening liver dysfunction, related to the embolic effect of TACE on an already compromised hepatic vascular supply. Sub-selective and super-selective TACE in selected patients with PVT is associated with overall survival of 7.4 to 10.2 months [43–46], only marginally better than systemic sorafenib, and inferior to survival that has been reported with other modalities, in particular SIRT, also known as TARE. This technique employs radioactive particles (Y90) which are directly injected in the hepatic arteries through super-selective catheterization, with a micro-embolic effect [47]. Both commercial products currently available (SIR Spheres and Theraspheres) are considered permanent embolic agents, even if their smaller size have much less embolic effect than TACE, with less effect on hepatic vascular dynamics [48]. The radiation activity may be delivered to the whole liver, to both lobes sequentially, to a single lobe, or to a segment. TARE has found application as a loco-regional therapy for unresectable HCC that is not amenable to TACE because of diffuse or multifocal disease, or as an alternative to TACE in selected patients [49]. Only recently its unique role in PVT has been definitely assessed, with a progressive shift from TACE [32].

Recently, two trials comparing SIRT to sorafenib in advanced HCC (SARAH trial [50] and the SIRveNIB trial [51]) were designed to prove the superiority of SIRT over sorafenib, but failed to meet their primary endpoint: median overall survival was comparable between both treatment groups: 8.0mo with SIRT compared to 9.9mo with sorafenib in the SARAH trial (p = 0.179), 8.8mo with SIRT compared to 10mo with sorafenib in the SIRveNIB trial (p = 0.36). However, in both studies response rate was significantly higher in the SIRT as compared to sorafenib group (roughly 20% vs 12% in the treated population), suggesting that SIRT may be more likely to control the tumor within the liver [52].

Although no randomized controlled trial has directly compared TARE with TACE, numerous series have reported favorable outcomes and acceptable safety profiles in HCC patients [53–57]. The largest group of 291 PVT patients treated with glass-based Y90 microspheres (TheraSphere), reported by Salem and colleagues [55], showed overall survival of 16.6mo among Child-Pugh A cirrhotics with branch PVT, decreasing to 4.5mo among Child-Pugh B cirrhotics with main PVT. Differently from this study, all patients in our series were treated with resin-based microspheres (SIR-Spheres), showing no significant difference comparing the survival curves of patients without PVT and patients with portal invasion, whether or not the vein occlusion was in the main portal trunk or in a portal branch.

In this study we present a large series of advanced-stage HCC patients receiving TARE, with the goal of understanding whether and how the presence of PVT alters survival in BCLC Stage C HCC after Y90 radio-embolization. Patients in our series were sorted by different baseline PVT characteristics to build and compare Kaplan-Meier survival curves during 36-month follow-up. The absence of significant statistical difference in the comparison of patients with PVT (main trunk and/or peripheral branch) and those with no sign of portal invasion and in the comparison of PVT patients with main trunk versus peripheral branch obstruction was the most surprising result. Even the comparison of survival curves for patients with main trunk PVT and those with no portal invasion showed no significant difference, thus meaning that PVT did not significantly affect survival after TARE. In particular, these results differentiate our investigation from a previous multicenter study published by Sangro et al. [56] in which a significant difference in survival was established between patients with portal branch and main trunk portal invasion. This difference was not attributable to the device, which was resin-based SIR Spheres in both studies.

Differently from previous studies focused on HCC survival after TARE, patients in our series showed higher response rates, especially in comparison with large randomized studies such as SARAH and SIRveNIB whose response rate (partial response + complete response) was up to 25%, against almost 70% at three months and 90.5% at nine months in our study. This was probably due to the different study design (randomized vs. non-randomized) and/or to patient recruitment, due to the strict inclusion and exclusion criteria of our study.

Another crucial point in our analysis was the high regression rate of portal thrombosis in patients still alive at T_2_ (13/27, 48.1%), with the maximum effect in retracting portal thrombus registered in the time-range T_0_-T_2_. It is rational to postulate that PVT regression delay is due to the internal radiation brachiterapic effect of Y-90 on the tumoral tissue of the thrombus, which has been proved to continue over months after the treatment [58]. Future studies should investigate whether PVT regression could represent a valuable marker of TARE clinical success and a prognostic indicator of improved survival.

The lack of severe (grade >3) adverse events according to CTCAE after TARE in a cohort with a high incidence of cirrhosis was another unexpected outcome, rarely occurred in previously published papers on this topic. Even this result is probably due to the strict patient selection.

The main limitations of this study are represented by the single-center nature and the non-randomized design.

In conclusion, our results provide a sound indication that TARE is safe and efficient in patient with advanced-stage HCC (classified as Stage C according to BCLC), even in case of PVT occurrence. Indeed, PVT did not affect survival. The location of the thrombus (portal branch vs. main trunk) seemed not to be relevant in our series. Moreover, thrombus regression was obtained in most cases and could be considered as a valuable predictor of procedure success, although a multicenter study on a larger cohort of patients is needed to establish its sensitivity and specificity.

## Supporting information

S1 Table Patients table for recruitment and statistical analysis. All data used in our statistical analysis are in this table, included patients characteristics, pre-treatment clinical and laboratoristic assessment, and follow up. All data underlying the findings described in this manuscript are contained in this table (S1 Table) and are fully available without restriction.
(XLS)

## Abbreviations

HCC: Hepatocellular Carcinoma
PVT: Portal Venous Thrombosis
BCLC: Barcelona Clinic Liver Cancer
mRECIST: modified Response Evaluation Criteria in Solid Tumors
EASL: European Association for the Study of the Liver
AASLD: American Association for the Study of Liver Disease
SHARP: Sorafenib HCC Assessment Randomized Protocol
ENRY: European Network on Radioembolization with Yttrium 90 Resin Microspheres
AFP: Alpha-fetoprotein
TACE: Trans-arterial Chemo-embolization
cTACE: conventional TACE
TAELE: Trans-arterial Ethanol-Lipiodol Embolization
TARE: Trans-arterial Radio-embolization
SIRT: Selective Internal Radiation Therapy
Y90: Yttrium-90
MDCT: Multi Detector Computed Tomography
TNM: Tumor-Node-Metastasis classification system
ECOG: Eastern Cooperative Oncology Group
^99m^Tc-MAA: Technetium-99 m-labelled macro-aggregated albumin
BSA: Body Surface Area
CTCAE: National Cancer Institute’s Common Terminology Criteria for adverse events
